# Severe Pulmonary Hypertension as Initial Presentation of SLE: A Case Report and Literature Review

**DOI:** 10.1155/2020/6014572

**Published:** 2020-05-15

**Authors:** Rabia Kiani, Muhammad Danial Siddiqui, Hamza Tantoush

**Affiliations:** Department of Internal Medicine, University of South Dakota, Vermillion, SD, USA

## Abstract

Severe pulmonary artery hypertension (PAH) is a rare initial presentation of systemic lupus erythematosus (SLE). SLE associated with PAH carries worse prognosis that isolated SLE. However, there has been improvement in mortality of the patients in the recent years owing to newer treatment options available. Early recognition remains of prime importance. We present here a case of young female who presented with severe pulmonary hypertension with right heart failure leading to cardiogenic shock and was found to have SLE. She was started on appropriate treatment; however, given the severity of her illness, the patient did not survive. This case highlights the importance of early recognition and prompt treatment of SLE-associated PAH, which might improve the survival rate in the patients.

## 1. Introduction

Systemic lupus erythematosus (SLE) is an autoimmune disease involving multiple organ systems. The range of clinical manifestations can vary from mild musculoskeletal disease to life-threatening renal, central nervous system, respiratory, and hematological system involvement. With the advancement in diagnostic and treatment strategies for SLE, the mortality has greatly improved [1]. However, it remains relatively higher for SLE associated with pulmonary artery hypertension (PAH).

Pulmonary hypertension (PH) is a heterogeneous group of disorders that carries poor prognosis leading to right heart dilatation and failure. It is defined as mean pulmonary artery pressure >25 mmHg at rest measured during right heart catheterization [2]. The World Health Organization (WHO) has classified PH into 5 different categories based on etiologies and pathophysiology. Category 1 includes pulmonary arterial hypertension (PAH), which is composed of different groups of disorders categorized as idiopathic and familial and associated with other disorders (e.g., connective tissue diseases (CTD)) [3]. Systemic sclerosis is considered the most common cause of PAH; however, SLE is increasingly recognized as emerging cause among CTD patients. The prevalence of PAH ranges from 0.5% to 43% in SLE [4]. Severe PAH is rarely seen as an initial presentation of SLE. We present here a case of young healthy women who presented to the hospital with severe PAH leading to right heart failure and cardiogenic shock, as the sole initial presentation of SLE.

## 2. Case Presentation

A 32-year-old female patient who initially presented to her primary care physician with complaints of progressively worsening shortness of breath (SOB) on exertion and bilateral lower extremity edema for a duration of 2 months. She also endorsed fatigue during that time; however, she denied any fevers, chills, orthopnea, joint pains, myalgias, or arthralgias. She did notice occasional chest pain with exertion for a similar period. Her past medical history included hypertension for which she was recently started on losartan. She also reported a history of sinus infection two months back which was treated with antibiotics. Physical examination showed mild bilateral pitting edema in lower extremities, no jugular venous distension, regular rhythm with no murmurs appreciated, and bilateral air entry in the lungs. There was no evidence of peripheral cyanosis, arthritis, rash, jaundice, or skin telengectasias. Initial workup showed hemoglobin 13.8 g/dL, hematocrit 41.1%, white blood cell count 2.9 K/*μ*L (lymphocyte 0.4 K/*μ*L, reference range 0.8–4.1 K/*μ*L), platelet count 139 K/*μ*L, BNP 482, CRP 9.7 mg/L (reference range <5.0 mg/L), and ESR 54 mm/HR (reference range 0–20 mm/HR). There was evidence of proteinuria (3+) on her urinalysis. sGOT, sGPT, LDH, and creatinine were normal. Chest x-ray was remarkable for interstitial infiltrates concerning for pulmonary edema and cardiomegaly. CT chest without contrast showed moderate to large pericardial effusion, enlargement of the central pulmonary arteries with the pulmonary trunk measuring 3.4 cm, bilateral axillary lymphadenopathy, and 3 mm noncalcified pulmonary nodules in the right upper lobe and left upper lobe. Given the worsening shortness of breath, low blood pressure, and pericardial effusion seen on CT scan, the patient was admitted to the hospital.

**Figure 1:**
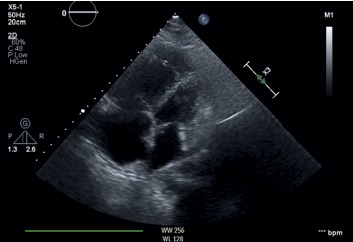
Echodcardioraphy apical 4 chamber view showing right atrial and right ventricular dilation.

**Figure 2:**
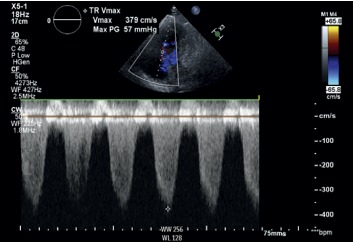
Continuous wave-Doppler study showing tricuspid insufficiency with an estimate peak pressure gradient of 57 mm Hg.

On admission, echocardiogram was obtained which showed an ejection fraction (EF) of 55–60%, severe right ventricular and right atrial dilation, pulmonary artery systolic pressure (PASP) of 81 mmHg, and pericardial effusion without tamponade physiology. Cardiothoracic surgery team was consulted, and the patient underwent pericardial window during which 300 ml of serous fluid was drained. Soon after the procedure, she developed hypotensive shock and became unresponsive. She was intubated for airway protection and started on norepinephrine along with dobutamine for inotropic support for the right ventricle. She was transferred to the intensive care unit (ICU) for suspected cardiogenic shock (CS). The patient underwent urgent right heart catheterization (RHC), which showed severe pulmonary artery hypertension (PAH) with pulmonary artery pressure (PAP) of 97/52 mmHg, normal pulmonary artery wedge pressure (PAWP) of 14 mmHg, and reduced cardiac output (CO) of 3.61 L/min, thereby confirming primary PH (Table 1). Pericardial fluid analysis/biopsy was negative for infection as well as malignancy but showed signs of chronic inflammation. Given new onset right heart failure, no clear etiology of her symptoms, and her demographics, she underwent workup for connective tissue diseases. Initial autoimmune workup showed anti-nuclear antibody (ANA) titer of 1 : 640 (normal <1 : 40); a positive anti-double-stranded DNA, anti-SSA, anti-SSB, anti-Smith antibody; and decreased complement C3 and C4. Workup for anti-phospholipid antibody syndrome was unremarkable. SLE was suspected on the basis of proteinuria, lymphopenia, positive ANA, anti-Smith antibody, and anti-ds-DNA antibody (American college of Rheumatology Criteria of 1997). Diagnosis of SLE was established with a total SLEDAI score of 15 (0–150) (fever, hematuria, leukopenia, proteinuria, complement consumption, and increased anti-ds-DNA Abs). She was also found to be positive for anti-neutrophil cytoplasmic antibodies (ANCA+) with atypical perinuclear patterns (Figures 1 and 2).

Subsequently, the patient developed multi-organ failure (MOGF) secondary to CS from severe PAH (World Health Organization group 1) possibly related to newly diagnosed SLE-related connective tissue disease (SLE-CTD). With regards to PH severity, she fell in the criteria of WHO functional class IV, which includes symptoms at rest or symptoms with physical activity. Given this, her PH could be classified as group 1 PH, and she was started on prostacyclin infusion (Remodulin 19 mcg/kg/min) for functional class IV. Later on, nitric oxide (NO) was added for severe PAH. NO was titrated from 20 to 60 parts per million. In addition to vasopressor therapy, inotropic support, and continuous renal replacement therapy (CRRT), she was also started on solumedrol 500 mg IV BID and hydroxychloroquine 200 mg once daily by rheumatology for a diagnosis of SLE-CTD leading to PAH.

Despite all of this, the patient's mean PASP continued to remain high with PAP of 64/40, pulmonary vascular resistance (PVR) of 15 wood units, and cardiac index (CI) of 2.3. Her condition continued to deteriorate, and it was decided to transfer her to a facility capable of extracorporeal membrane oxygenation (ECMO) and heart-lung transplant evaluation. Due to concern of SLE immune complex disease in the setting of multi-organ failure and declining clinical status, it was decided that the patient should be started on therapeutic plasma exchange (TPE). Along with ECMO, the patient underwent seven sessions of therapeutic plasma exchange (TPE). She received cyclophosphamide 500 mg IV after the fourth session of TPE. After a few days of remaining on ventilator and aggressive therapies, there was no improvement in her clinical condition. It was decided that the patient was not a candidate for heart-lung transplant due to her critical condition and MOGF. This was discussed with the family who chose to withdraw cares. Soon thereafter the patient passed away.

## 3. Discussion

Pulmonary artery hypertension is a subclass of pulmonary hypertension (PH), characterized by the presence of precapillary pulmonary hypertension. It is defined by end-expiratory pulmonary artery wedge pressure (PAWP) as ≤15 mmHg and pulmonary artery resistance >3 Wood units on right heart catheterization. In addition, other causes of PH including left heart failure, primary lung disease, and venous thromboembolic disease need to be excluded.

Various retrospective analyses have reported variability in the prevalence of PAH associated with SLE (SLE-aPAH). Some older studies have noted the prevalence between 0.5% and 43% [5]. Arnaud et al. and Hass et al. noted the prevalence to be between 0.5% and 17.5%, as seen in two newer French studies [6, 7]. In one study of 288 patients with SLE, the point prevalence of PAH was noted to be 4.25%. In another study by Timothy et al., 28 patients were followed over a period of five years to determine the prevalence and progression of SLE-associated PAH. The study also enrolled 20 healthy individuals without cardiac or pulmonary disease as normal controls. At 5 year follow-up, the prevalence of PAH increased from 14% to 43% in the SLE group. There was a significant increase reported in pulmonary artery pressure from 23.4 vs 27.5 mm Hg in SLE patients (*p* < 0.005) [8]. The wide range in reported prevalence of PAH in SLE is likely due to factors including differences in cut-offs for pulmonary artery pressure (25 mmHg vs 30 mmHg), diagnostic methods (right heart catheterization vs transthoracic ECHO), and patients characters/ethnicity.

The pathophysiological mechanisms linking PAH to SLE are complex and still a subject of investigation. Various causative mechanisms have been proposed for SLE-aPAH with genetic predisposition, immune system dysfunction, and environmental stimuli (e.g., infections) playing a pivotal role. Various studies have proposed that an initial insult in the form of infections, hypoxia, wall stress, or unknown stimuli to endothelium leads to an imbalance between production of vasoconstrictors and vasodilators, with elevated levels of endothilin-1 and thromboxane A2, which are the major vasoconstrictors, seen in PAH. Also seen are decreased levels of vasodilator prostacyclin. This pulmonary vasoconstriction leads to production of hypoxia inducible factor and erythropoietin, which leads to proliferation of smooth muscles in pulmonary vessels and remodeling of vasculature [9]. Another mechanism includes deposition of immune complexes and complements in the pulmonary vessels, leading to activation of inflammatory cells and release of inflammatory cytokines. This leads to endothelial damage and further vascular remodeling [10]. Another contributing process is recurrent thromboembolic disease particularly seen in patients with positive anti-phospholipid antibodies leading to hypercoagulable state. In summary, a combination of vasoconstriction, vessel wall remodeling, and in situ thrombosis underlie the complex pathophysiological pathway that leads to increased pulmonary artery pressure.

Since presence of PAH carries worse prognosis in SLE patients, prompt recognition and early treatment initiation is of utmost importance. Three main molecular pathways are targeted in the treatment of SLE-aPAH: the nitric oxide (NO) pathway, the endothilin-1 pathway, and the prostacyclin pathway. NO is produced by endothelin cells and exerts its vasodilatory effect by causing relaxation of vascular smooth muscles. This effect is mediated by intracellular cyclic GMP. This pathway is targeted by phosphodiesterase (PDE) inhibitors, which prevent PDE-mediated destruction of cGMP. Other agents in this class are cGMP stimulators. Endothelin is a potent vasoconstrictor that is overexpressed in PAH, and endothelin receptor antagonists target this pathway. Prostacyclin I2 causes conversion of AMP to cyclic AMP (cAMP), which caused vasodilation of vascular smooth muscles. Prostanoids or prostacyclin I2 analogues activate adenylate cyclase mimicking prostacyclin I2 function. These classes of drugs are FDA approved only for PAH, not in other WHO classes of pulmonary hypertension [5].

In treatment of SLE-aPAH, therapeutic decisions are based on variable factors including echocardiography, WHO classification functional class (FC), exercise capacity, and hemodynamic and laboratory parameters. A combination of immunosuppressants and pulmonary vasodilators is mostly employed depending on disease severity. Patients with less severe PAH at baseline, as evident by right heart catheterization and better functional class (FC I–II), can be treated with immunosuppressants alone, a combination of cyclophosphamide and prednisone. Most patients with WHO FC I should be initially monitored for development of any PAH-associated symptoms. In addition, conditions classified as risk factors for development for PH should be treated aggressively. Multiple oral therapies have been approved for treatment of WHO FC II with symptoms. No direct trials have been conducted so far to support the superiority of one treatment agent over the other. These include 3 orally active endothelin receptor antagonists (ETRA) (bosentan, ambrisentan, and macitentan), 2 orally active PDE5 inhibitors (sildenafil and tadalafil), and 1 orally active guanylate cyclase stimulator (riociguat). If no improvement is seen with single agents, incremental doses and combination therapies are recommended. For FC III, similar agents alone or in combination are recommended initially. However, patients with FC III with rapid progression of their disease or other markers of poor prognosis are candidates for initial therapy with parenteral prostanoid agents. These agents are also considered for treatment if patients with FC II or III show disease progression despite use of the first line oral medications or intolerance or side effects to oral therapies. For patients with FC class IV, treatment with intravenous (IV) prostenoid as an initial agent is recommended. Inhaled prostanoid can be used in place of IV, in patients unable to manage IV medication. These can be used in combination with ETRA in FC IV and in FC III with disease progression [11, 12]. Patients with better functional class and higher disease activity for SLE (and positive anti-ds-DNA and anti-Sm antibody) have shown to respond well to immunosuppression alone [5].

Our patient presented with WHO FC III-IV functional status and was found to have moderate to severe pericardial effusion and severe pulmonary artery hypertension. She was started on high-dose steroids along with Plaquenil and prostacyclin once diagnosis of SLE was confirmed. She was transferred to center with expertise in management of SLE-aPAH, where above therapies were continued. However, given the severity of her disease, family chose for comfort care and the patient unfortunately passed away.

SLE associated with PAH carries worse prognosis than isolated SLE. There is paucity of literature regarding the survival of patients with SLE who present initially with PAH or patients with severe PAH. In a study of 20 patients, 55% of whom received advance therapy for PAH, and the median survival was 13 months [13]. The 5-year survival in these patients is 60.2% compared to 97.8% in isolated SLE [14]. Although SLE-aPAH carries poor prognosis, with the development of newer therapeutic options, the median survival in these patients has improved from less than 3 years to a 2-year survival of more than 90% [15].

## 4. Conclusion

Severe PAH is a rare initial presentation of SLE. The prognosis in these patients is very grim. Advancement in therapeutic options has improved survival; however, the mortality is still very high. Prompt recognition remains of utmost importance as early institution of treatment might improve the survival in these patients.

## Figures and Tables

**Table 1:** Hemodynamic parameters from right heart catheterization.

Hemodynamic parameters (units)	Reference range	Current PAH exacerbation
RAP (mmHg)	2–6	RAP 17/16
PAWP (mmHg)	4–12	14
PAP (mmHg)	20–30 systolic	97/52
	8/12 diastolic	
Mean PAP (mmHg)	25	69
CO (L/min)	4–8	3.61
CI (L/min/m^2^)	2.5–4	2.03
PVR (woods unit)	0.5–1.1	15

RAP = right atrial pressure, PAWP = pulmonary artery wedge pressure, PAP = pulmonary artery pressure, CO = cardiac output, CI = cardiac index, PVR = pulmonary vascular resistance.
